# Triple-negative expression (ALDH1A1-/CD133-/mutant p53-) cases in lung adenocarcinoma had a good prognosis

**DOI:** 10.1038/s41598-022-05176-0

**Published:** 2022-01-27

**Authors:** Naoki Yamashita, Tetsuya So, Takeaki Miyata, Takashi Yoshimatsu, Ryuji Nakano, Tsunehiro Oyama, Wataru Matsunaga, Akinobu Gotoh

**Affiliations:** 1 Department of Education for Medical Research Base, Hyogo College of Medicine, 1-1 Mukogawa-cho, Nishinomiya, 663-8501 Japan; 2 Department of Thoracic Surgery, Shin-Komonji Hospital, Fukuoka, Japan; 3 Department of Thoracic Surgery, Shin-Kuki General Hospital, Saitama, Japan; 4 Department of Thoracic Surgery, Fukuoka-Wajiro Hospital, Fukuoka, Japan; 5 Department of Pathology, Fukuoka-Wajiro Hospital, Fukuoka, Japan; 6 Imamitsu Home Care Clinic, Kitakyusyu, Japan

**Keywords:** Cancer, Stem cells, Oncology

## Abstract

Cancer stem cells (CSCs) are major contributors to the malignant transformation of cells because of their capacity for self-renewal. Aldehyde dehydrogenase1A1 (ALDH1A1) and CD133 are promising candidate of CSC markers in non-small cell lung cancer (NSCLC). Furthermore, TP53 is frequently mutated in lung cancer, and the loss of its function is associated with malignant characteristics. However, the relationship between CSCs and mutant p53 in lung adenocarcinoma is not well-established. We examined the expression of ALDH1A1, CD133, and mutant p53 in lung adenocarcinoma patients and conducted a clinicopathological study. Triple-negative cases without ALDH1A1, CD133, and mutant p53 expression in lung adenocarcinoma were shown to have a much better prognosis than others. Our present results suggest that detection of CSC markers and mutant p53 by immunohistochemical staining may be effective in therapeutic strategies for lung adenocarcinoma.

## Introduction

The recurrence rate of lung cancer is as high as 80% even in early stages that are treated with chemotherapy^1^, and almost one-quarter of all cancer-related deaths are due to lung cancer^2^. A major clinical problem associated with lung cancer is the acquired resistance of tumors to chemotherapy^3^, with NSCLC patients having a 5-year survival rate of less than 20%^4^. In clinical practice, approximately 20% of NSCLCs are operable, but the 5-year survival rate remains low despite advances in chemoradiotherapy, targeted therapy, and immunotherapy for inoperable cases (7%–20%), and the recurrence rate remains high in 30%–50%^5^. From these knowledges, novel therapeutic strategies are required to overcome cancer recurrence, metastasis, and resistance to chemo- and radiotherapy.

CSCs are an exceedingly rare population among the entire cancer cell population (less than 1% for most solid tumors) that exhibits high tumorigenicity^6^. The main CSC properties are as follows: (a) unique self-renewal ability to produce the daughter cells with the same stem cell characteristics (similar to normal stem cells); (b) the ability to differentiate into a variety of cancer cell lines and promote cell proliferation and overall tumor survival; and (c) high tumorigenic potential to expand and create non-CSC strains and form new tumors^7^. In reality, the characteristics of CSCs include self-renewal, differentiation capability, high infiltration and migration properties, high tumorigenicity, and resistance to chemotherapy^8–11^. Accumulating evidence supports the existence of a CSC phenotype in human lung cancer, and several CSC markers, such as ALDH1A1, CD133, and CD44, have been characterized in lung cancer^12,13^.

ALDH1 proteins (mainly ALDH1A1, ALDH1A2, and ALDH1A3) are primarily localized in the cytosol of cells in various tissues and let aliphatic aldehyde oxidize at retina. Member of the ALDH family has a main biological role in cell protection through the detoxification of aldehydes, but they also regulate cell proliferation, differentiation, and survival^14^. And especially, ALDH1A1 is a putative hematopoietic stem cell marker associated with drug resistance that increased of many cancer types^15^.

CD133 antigen, also known as prominin-1, is a member of the pentaspan transmembrane glycoprotein family that is specifically located to cellular protrusions. In fact, it is widely utilized as a CSC biomarker in various types of cancer, including liver, stomach, kidney, and lung cancer^16–19^.

We previously reported that lung adenocarcinoma that is negative for both CD133 and ALDH1A1 had a significantly better prognosis regarding both overall survival (OS) and disease-free interval (DFI)^20^. Furthermore, we reported that patients with stage I + II lung adenocarcinoma who were negative for CD133 had a significantly better DFI^21^. Thus, there is certainly a close correlation between CSCs and lung adenocarcinoma.

*TP53*, one of tumor suppressor genes, has some functions in gene transcription, cell division, and DNA repair^22–24^. Conversely, *TP53* gene mutations are the most common genetic abnormalities in human malignancies and are also frequently detected in primary lung cancer; mutant p53 is observed in 70% of small cell lung cancers and 47% of non-small cell lung cancers^25^. In fact, cases of surgically resectable non-small cell lung cancer (NSCLC) with mutant p53 have a significantly poorer prognosis, and it has been reported that mutant p53 can be an independent prognostic factor^26^.

To date, there have been many reports about CSCs and p53, but few have investigated the association between CSCs and p53 in lung cancer. Here, we scrutinized the expression and prognosis of CSC marker-positive cells locally in resected samples from patients with lung adenocarcinoma and then explored the association between CSC markers and mutant p53 expression.

## Results

Of 286 patients with lung adenocarcinoma, 194 (67.8%) were eligible for this study. The clinicopathological parameters are shown in Table 1. OS and DFI were poor in older patients (≥ 75 years) (*p* < 0.05). In terms of sex, OS was much better in women (n = 88, 45.4%) than in men (*p* < 0.05), but there was no significant difference in DFI. Current smokers (n = 79, 34.5%) had significantly worse OS and DFI than never and former smokers (*p* < 0.05). In terms of tumor markers, patients with high serum CEA levels (> 5 ng/ml) had significantly worse OS and DFI compared with those with normal CEA levels. Partial resection (n = 25, 13.3%) conferred a worse prognosis than complete resection, and patients with advanced-stage lung adenocarcinoma had a worse prognosis than patients with early-stage disease (*p* < 0.05). However, we could not obtain a clear association between each parameter and CSC or mutant p53 (data not shown).

**Table 1:**
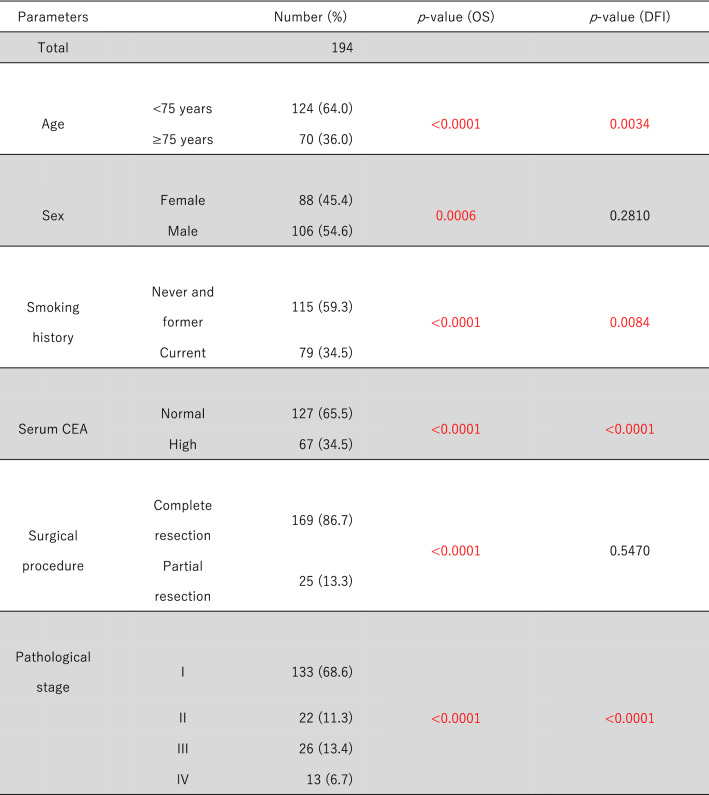
Clinicopathological parameters and prognosis.

**Figure 1:**
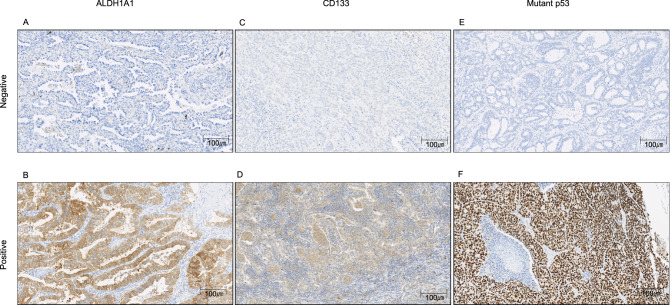
Immunohistochemical staining of ALDH1A1, CD133, and mutant p53 of samples from lung adenocarcinoma patients. ALDH1A1 staining intensity was rated as weak (1+), moderate (2+), or strong (3+) and multiplied by the percentage of positive cells. ALDH1A1 score = (% of cells of intensity 1 × 1) + (% of cells of intensity 2 × 2) + (% of cells of intensity 3 × 3). The total scores were categorized as follows: 0–100 = grade 1; 101–200 = grade 2; and 201–300 = grade 3. Grade 2 or 3 tumors were considered positive for ALDH1A1. (**A**) ALDH1A1 score = 0 (intensity 0 × 0% positive cells). (**B**) ALDH1A1 score = 220 (intensity 1 × 20% positive cells + intensity 2 × 30% positive cells + intensity 3 × 50% positive cells). The percentage of CD133- and mutant p53-positive cells was graded as 0%–100%. CD133 positivity was defined as staining of more than 20% of the tumor cells (negative: ≤ 20%; positive: > 20%). (**C**) CD133-positive cells were 0%, negative. (**D**) CD133-positive cells were 100%, positive. (**E**) Mutant p53-positive cells were 0%, negative. (**F**) Mutant p53-positive cells were 100%, positive.

Regarding CSCs, Fig. 1 shows the immunohistochemical staining of ALDH1A1, CD133, and mutant p53 in sections of resected samples. Table 2 shows the negative and positive rates of CSCs and mutant p53, as well as the significant differences between OS and DFI.

**Table 2:**
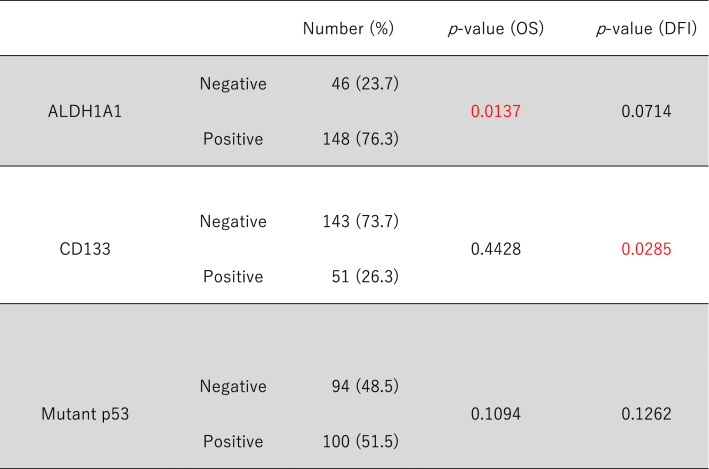
Results and prognosis of immunohistochemical staining of CSCs and mutant p53.

**Figure 2:**
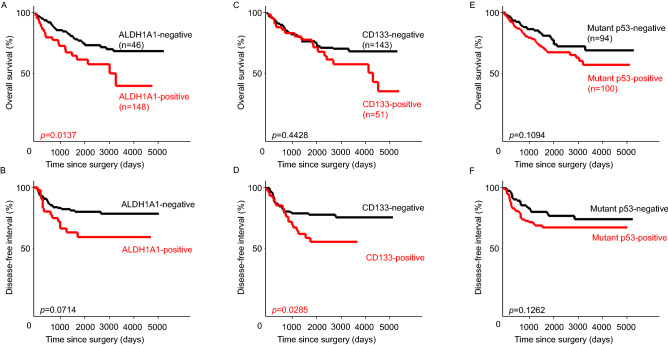
Kaplan–Meier curves of overall survival (OS) and disease-free interval (DFI) of ALDH1, CD133, and mutant p53. (**A**) OS curves stratified by ALDH1A1 expression. (**B**) DFI curves stratified by ALDH1A1 expression. (**C**) OS curves stratified by CD133 expression. (**D**) DFI curves stratified by CD133 expression. (**E**) OS curves stratified by mutant p53 expression. (**F**) DFI curves stratified by mutant p53 expression.

Approximately 23.7% (n = 46) of patients were ALDH1A1-positive. OS of the ALDH1A1-positive group was significantly worse than that of the ALDH1A1-negative group, but there was no difference in DFI (Fig. 2A,B).

Approximately 26.3% (n = 51) of patients were CD133-positive, and the DFI of the CD133-positive group was significantly worse than that of the CD133-negative group, but there was no difference in OS (Fig. 2C,D).

Although approximately 51.5% (n = 100) of the patients were in the mutant p53-positive group, there was no significant difference in OS or DFI compared with the mutant p53-negative group (Fig. 2E,F).

**Figure 3:**
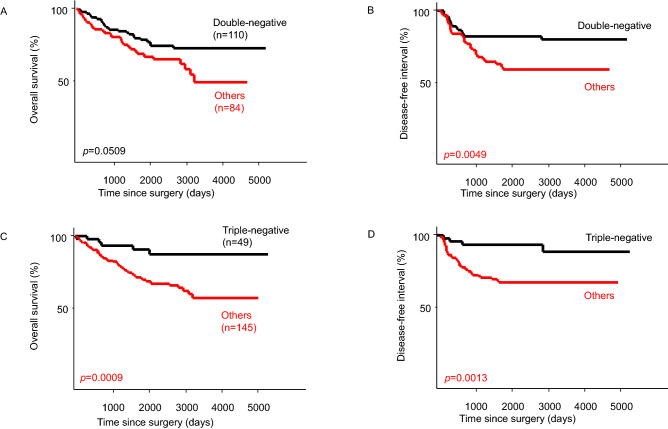
Kaplan–Meier curves of overall survival (OS) and disease-free interval (DFI) of the double-negative (ALDH1A1-negative + CD133-negative) and triple- negative groups (CSC marker-negative + mutant p53-negative). (**A**) OS curves stratified by the double-negative group or others. (**B**) DFI curves stratified by the double-negative group or others. (**C**) OS curves stratified by the triple-negative group or others. (**D**) DFI curves stratified by the triple-negative group or others.

The phenotypes of CSCs and mutant p53 were also evaluated. The group negative for both ALDH1 and CD133 (double-negative group) had a significantly better prognosis than the other groups regarding DFI, but no significant difference in OS was observed (Fig. 3A,B). In patients negative for CSC markers and mutant p53 (triple-negative group), the prognosis was significantly better than that of the other groups in terms of both OS and DFI (Fig. 3C,D).

## Discussion

In this study, we revealed that cases expressing ALDH1A1 had much worse OS than ALDH1A1-negative cases when ALDH1A1 alone was examined, but there was no significant difference in DFI. ALDH (a detoxifying enzyme that oxidizes intracellular aldehydes) is a member of a group of enzymes that protect stem cells from oxidative damage by causing the oxidation of aldehydes to carboxylic acids^27^. So far, ALDH1 expression has been assessed for the identification of CSCs in several cancer types including leukemia and breast, neural, head and neck, colon, liver, and lung cancer^28^. In addition, ALDH1 converts retinol to retinoic acid, which causes stem cell differentiation and proliferation in the early stages^29^. Furthermore, ALDH1A1 expression has been shown to be related with resistance to chemotherapy^30,31^. Finally, a recent meta-analysis revealed that increased ALDH1A1 expression is associated with poor OS and disease-free survival in lung cancer patients^32^.

In the current study, we revealed that CD133-expressing cases had a much worse DFI than CD133-negative cases when CD133 alone was investigated, but there was no significant difference in OS. CD133 is an 865-amino acid penta-span transmembrane protein that has been accepted as a principal marker of stemness in several solid tumors^33^. Some previous studies have shown that human lung cancer contains CD133-positive CSCs that can self-renew and have high tumorigenicity^34^. In addition, the expression of CD133 in NSCLC is associated with the degree of cell differentiation, lymph node metastasis, and prognosis^35^. Furthermore, CD133 expression is negatively correlated with the prognosis of patients with lung cancer because lung tumors containing CD133-positive cells are resistant to cisplatin^36^. Therefore, CD133 expression is a marker for lung CSCs. Our previous study revealed that immunohistological CD133 expression was correlated with the pathological stage of human adenocarcinoma, especially stage I + II disease. Apart from these, various studies suggested that CD133 may have an important role in regulating the expression of CSC genes by interacting with several signaling pathways. However, further investigations are necessary to understand possibility of CD133 in CSC regulation^37^.

In this study, there was no clear significant difference in mutant p53 expression between OS and DFI, but mutant p53-expressing cases tended to have a poorer prognosis than negative cases. *TP53* encodes the protein consisting of 393 amino acids (p53) in short arm of chromosome 17, which acts as a transcription factor that regulates the expression of other genes. And wild-type p53 has the role of suppressing the accumulation of mutant cells through the induction of apoptosis, giving anticancer drug sensitivity and radiosensitivity^38,39^. Furthermore, the DNA binding site of p53 is a hotspot for point mutations that result in changes to its three-dimensional structure that inhibit the binding of p53 to its target DNA, and loss of transcriptional activity indicates loss of its tumor suppressor function^40,41^. To date, various studies have revealed that abnormal p53 expression may promote the initiation and progression of CSCs^42,43^, and activation of mutant p53 was found to increase tumorigenicity by promoting symmetric self-renewal division and inhibiting macrophage accumulation^44^. Although these studies suggested that p53 could be a barrier to CSC formation, the precise mechanism by which p53 regulates CSC survival and tumorigenesis remained unclear. Other than the above, Steels et al. meta-analyzed that p53 expression is a negative prognostic factor for survival in NSCLC, regardless of the antibody used^45^. In addition, somatic mutations of *TP53* occur frequently during the development of human neoplasia, and because mutant p53 proteins are often much more stable than wild-type p53 protein, mutant p53 accumulates to a high level^46^. Furthermore, the expression of mutant p53 in serum was found to significantly reduce the survival rate of cancer patients. In this meta-analysis, p53 antibodies for cancer patients can predict worse outcomes, and mutant p53 levels in serum may be useful for future therapies^47^.

Various studies have shown the association between ALDH and CD133, including the following: NSCLC cells with relatively high ALDH1 activity are characterized by their increased ability to proliferate, self-renew, differentiate, and express CD133 CSC marker^48,49^. Furthermore, Jiang et al. found that ALDH expression was associated with the decreased survival of patients with stage I NSCLC and reported a high association between CD133 and ALDH1 expression. This suggests that these proteins are markers for the same tumor cell population^50^. In addition, double-positive cases of ALDH1 and CD133 were found to be expressed only in lung adenocarcinoma and squamous cell carcinoma, and were closely related to tumor type^51^. This suggests that CSCs may have varying importance in the development and progression of lung cancer, depending on the tumor type.

Regarding the association between CSCs and p53, a study by Hilla et al. showed that overexpression of ALDH1A1 in colorectal cancer was associated with reduced apoptosis, which indicated the involvement of ALDH1A1 in the mediation of mutant p53-dependent chemotherapy resistance^52^. An in vitro study, wild-type p53 was also described to suppress CD133 expression transcriptionally in colon cancer. In addition, the tumor-suppressive effect of wild-type p53 in some cancer cell lines needed p53-mediated CD133 inhibition^53^. These results guessed that CD133 may be a potential target for tumor inhibition in highly tumorigenic cancers with impaired p53 function^54^.

In this study, double-negative (ALDH1A1−/CD133−) cases had a much better DFI than others, but there was no significant difference in OS. Furthermore, triple-negative (ALDH1A1−/CD133−/p53−) cases had a notably better prognosis than other cases. However, there were several limitations to this study. First, this retrospective study is susceptible to bias. Second, in this study, additional adjuvant and systemic chemotherapy was given after surgery. Third, patient follow-up was not uniform, and differences in survival probability may have been influenced by the duration of the study. These factors may have influenced the resulting data, in a view similar to Sobhani et al.^47^.

In conclusion, lung adenocarcinoma negative for mutant p53 was presumed to have a good prognosis, but when the expression of mutant p53 was investigated in combination with CSC markers, the prognosis of these patients was better with statistical significance. These results suggest that mutant p53 expression may promote the expression of CSC markers and CSC activity in lung adenocarcinoma. Thus, CSC markers and mutant p53 may be effective targets in therapeutic strategies for lung adenocarcinoma. However, these functions have not yet been clarified completely and require further research. In future studies, we will elucidate the detailed mechanism involved and verify other CSC markers.

## Methods and materials

A total of 286 patients with lung adenocarcinoma who underwent complete resection or other surgery were investigated: 100 cases at Fukuoka-Wajiro Hospital from 2006 to 2012 and 186 cases at Shin-Komonji Hospital from 2010 to 2018. Clinicopathological parameters were assessed in pathological specimens. The mean age at the time of surgery was 70 years (range 40–92 years). None of the patients received chemotherapy or radiation before surgery. Tumor staging was performed in accordance with the 8th edition of TNM classification by the International Association for the Study of Lung Cancer (IASLC). The clinicopathological parameters were examined age, sex, smoking history, tumor marker level (carcinoembryonic antigen, CEA), surgical procedure, and pathological stage.

### Staining for markers

A paraffin-embedded section was prepared from the resected lung sample, and histological diagnosis was obtained by hematoxylin–eosin (HE) staining. Sectioning of lung adenocarcinoma samples was donated for immunohistochemical staining of ALDH1A1, CD133, and mutant p53 using a standard immunoperoxidase technique, as described previously^20^.

### Evaluation of immunohistochemical staining

ALDH1A1 staining was performed on 4-μm-thick paraffin sections using a mouse monoclonal antibody (anti-ALDH1A1; ab52492; Abcam, Cambridge, MA, USA) at a 1:100 dilution. As previously reported, the results were semi-quantitatively graded on the basis of the percentage of stained cells and the staining intensity^20^.

CD133 staining was performed on 4-μm-thick paraffin sections using a mouse monoclonal antibody (anti-CD133; MAB4399-I; Millipore, Temecula, CA, USA) at a 1:200 dilution. As previously reported, the CD133 expression score was defined as the proportion of cells with strong membranous staining in tumor sections^20,21^.

Mutant p53 staining was performed on 4-μm-thick paraffin sections using a mouse monoclonal antibody (anti-p53; M700129; Agilent., Santa Clara, CA, USA) at a 1:200 dilution. In this study, we counted tumor cytoplasm stained with IHC as mutant p53, as previously reported by Steels, E. et al. and Ramael, M. et al.^45,46^. The percentage of positive cells was graded as 0%–100%. Mutant p53 positivity was defined as staining in more than 20% of the tumor cells (negative, ≤ 20%; positive, > 20%).

### Statistical analysis

Stained sections were evaluated using virtual slides (Nano Zoomer-XR, Hamamatsu Photonics Co., Ltd.). One physician with experience in pathological research and two physicians with experience in clinical research evaluated the specimens and derived the mean value. All data were analyzed using the statistical software StatView (SAS Institute Inc. Cary, NC, USA). The survival curves were evaluated using the Kaplan–Meier method. *P*-values ≤ 0.05 were considered statistically significant.

This study received ethical approval for human subjects from the Shin-Komonji hospital’s research ethics committee. Informed consent was obtained from each patient. Also, all methods were carried out in accordance with relevant guidelines and regulations.
